# Prevalence of Provocative Seizures in Persons with Epilepsy: A Longitudinal Study at Khon Kaen University Hospital, Thailand

**DOI:** 10.1155/2015/659189

**Published:** 2015-11-12

**Authors:** Nutthaya Vongkasamchai, Sunee Lertsinudom, Acharawan Topark-Ngarm, Udomlack Peansukwech, Kittisak Sawanyawisuth, Somsak Tiamkao, Integrated Epilepsy Research Group

**Affiliations:** ^1^Division of Clinical Pharmacy, Faculty of Pharmaceutical Sciences, Khon Kaen University, Khon Kaen 40002, Thailand; ^2^Integrated Epilepsy Research Group, Khon Kaen University, Khon Kaen 40002, Thailand; ^3^Department of Medicine, Faculty of Medicine, Khon Kaen University, Khon Kaen 40002, Thailand; ^4^Research Center in Back, Neck, Other Joint Pain and Human Performance (BNOJPH), Khon Kaen University, Khon Kaen 40002, Thailand

## Abstract

*Background and Objective*. Provocative factors are one causative factor of seizure attacks in persons with epilepsy (PWE). There are limited data of prevalence and major provocative factors in Asian populations.* Methods*. This study was performed at the Epilepsy Clinic, Khon Kaen University Hospital. The patients who aged 15 years or over, who had been treated at least 3 months with at least one antiepileptic drug, and who were followed up for at least one year were included. Data of seizure control and triggers were collected retrospectively from medical records. Data analysis was performed to identify independent provocative factors.* Results*. A total of 382 PWE met the study criteria. The mean age was 40.4 ± 0.8 years. Approximately 44% of the patients had at least one provocative factor. By multivariate analysis, the independent provocative factors with the first three highest adjusted odds ratios were sleep deprivation (adjusted OR = 8.64, 95% CI 3.73–19.99), alcohol consumption (adjusted OR = 6.76, 95% CI 1.44–31.78), and feeling stressful (adjusted OR = 2.97, 95% CI 1.29–6.86).* Conclusion*. Almost half of seizure attacks may be caused by provocative factors in Thai PWEs and some factors may be preventable. Avoidance of these factors should be emphasized to epilepsy patients for improving clinical outcomes and quality of life.

## 1. Introduction

Epilepsy is a common neurological disease and requires long-term treatment. It is estimated that more than 50 million people worldwide are diagnosed with epilepsy; 85% of patients are in developing countries. Approximately 2.4 million new cases of epilepsy are diagnosed per year [1]. Only 10–40% of people with epilepsy (PWEs) can control their seizures resulting in an important public health issue.

Uncontrolled epilepsy may lead to accidents or poor psychosocial status which become health issues themselves [2]. PWEs are more likely to have depression, anxiety, suicidal thoughts, and sudden death [3, 4]. Other than antiepileptic drug compliance, provocative or precipitating factors may lead to seizure attacks or intractable epilepsy. These factors include physical, psychological, and environmental factors. Sleep deprivation, starvation, and financial debt were previously purposed as provocative factors in PWEs [5–7]. Most studies regarding provocative factors were conducted in the western countries. Here, this study aimed to unveil provocative factors for seizure in Thai PWEs in terms of enumerating these provocative factors and their prevalences. The results of this study may apply to other similar Asian populations.

## 2. Methods

This study was a longitudinal study conducted at the Epilepsy Clinic, Srinagarind Hospital, Khon Kaen University, Thailand. This facility is a tertiary-care teaching hospital serving the Thai population in the Northeastern, Thailand. The study duration was between January 1st, 2011, and December 31st, 2011.

The inclusion criteria were adult PWEs aged more than 15 years and treated at the Epilepsy Clinic for at least 3 months with at least one antiepileptic drug and one year of follow-up. Eligible patients were asked to participate in the study and signed informed consents prior to the enrollment. The study protocol was approved by the ethics committee for human research, Khon Kaen University.

Baseline clinical data, types of epilepsy, treatments, history of seizure control, and factors associated with the seizures were collected. “Seizure-free” was defined as no evidence of seizure attack as identified by patients or others. Provocative factors including physical, psychological, and environmental factors were also recorded. All factors were referred to as being relevant to the last three-month period prior to the last clinic visit.

Patients were divided into two groups by self-reported presence or absence of seizure attack. Baseline and clinical characteristics of patients were calculated by using descriptive statistics. Univariate logistic regression analyses were applied to calculate the crude odds ratios of an individual provocative factor for having led to a seizure attack. Factors were included in subsequent multivariate logistic regression analyses to identify independent provocative factors for having seizure attacks. Analytical results were presented as crude odds ratios (OR), adjusted OR, and 95% confidence intervals (CI). Data analyses were performed with STATA software (College Station, Texas, USA) and SPSS software (Chicago, Illinois, USA).

## 3. Results

There were 459 PWEs treated at the Epilepsy Clinic during the year of 2011. Of those, 382 PWEs met the study criteria (83.22%). The mean (S.D.) age of all patients was 40.35 (0.83) years. Male patients accounted for 48.95% and 39.79% of patients were single. The mean (S.D.) duration of epilepsy treatment in the clinic was 2.55 (1.11) years. The most common seizure type was generalized tonic clonic seizure (59.42%). There were 113 patients (29.58%) who reported no seizure attack since the last visit. For those who had seizure attacks, the average (S.D.) of seizure attack was 7.46 ± 0.69 times/month. There were 168 PWEs (43.97%) who reported having at least one provocative factor.

PWE had significant lower age (37 versus 43), lower age onset of epilepsy (28 versus 36), more proportion of complex partial seizure (46.84% versus 28.32%), and more provocative factors (Table 1). Univariate and adjusted odds ratio for seizure attack were shown in Tables 2 and 3. Complex partial seizure, feeling stressful, sleep deprivation, and alcohol consumption were independently associated with seizure attack (Table 3) with adjusted odds ratios of 1.87, 2.97, 8.64, and 6.76, respectively.

## 4. Discussion

At least 44% of PWEs reported provocative factors associated with seizure attacks. This number is similar to a previous study by Nakken et al. [8]. In this study, sleep deprivation was the most common provocative factor, while other previous studies indicated that stress was the most common one [5–7]. This finding may be explained by different time frames of the studies. Nowadays, there are several available activities during night time particularly online games or internet. These activities are available 24/7 and therefore may reduce sleep time and also alter sleep schedules. At present, sleep deprivation may be the most common provocative factor rather than feeling stressed as in the past.

After adjustment for several factors as shown in Table 3, sleep deprivation gave the highest adjusted odds ratio at 8.64, followed by alcohol consumption (6.76) and feeling stressful (2.97). da Silva Sousa et al. reported that significant provocative factors in PWEs were sleep deprivation and stress [5], while alcohol consumption has never been reported as the independent provocative factor in the literature [5, 7, 9, 10]. Complex partial seizure was confirmed here that it may be difficult to control [11] than other types of seizure. Age was a significant factor for seizure attack by univariate logistic analysis, but it was not the independent factor by multivariate analysis or after adjustment for other factors (Tables 2 and 3).

All eligible PWEs had long-term and regular follow-ups at least for one year. Data therefore were quite valid. On the other hand, these data may be applied only for those PWEs who had regular treatment and follow-ups.

In conclusion, almost half of seizure attacks may be caused by provocative factors and some factors may be preventable in Thai PWEs. Avoidance of these factors should be emphasized to epilepsy patients for improving clinical outcomes and quality of life.

## Figures and Tables

**Table 1:** Clinical features of persons with epilepsy (PWE) categorized by presence of seizure attacks.

Factors	Seizure attack *n* = 269	No seizure attack *n* = 113	*p* value
Male, *n*	128 (47.58)	59 (52.21)	0.41
Median age, years	37 (26–50)	43 (28–56)	0.01
Median age onset of epilepsy, years	28 (15–41)	36 (18–52)	0.01
Median weight, kg	60 (52–68)	60 (51–67)	0.98
Single status, *n*	115 (42.75)	37 (32.74)	0.06
History of drug allergy, *n*	48 (17.84)	19 (16.81)	0.88
Type of seizure, *n*			
Simple partial seizure	25 (9.29)	15 (13.27)	0.27
Complex partial seizure	126 (46.84)	32 (28.32)	<0.01
Generalized tonic clonic seizure	161 (59.85)	66 (58.41)	0.79
Absence seizure	15 (5.58)	4 (3.54)	0.40
Atonic seizure	1 (0.37)	0 (0)	0.99
Myoclonic seizure	3 (1.12)	0 (0)	0.55
Tonic seizure	3 (1.12)	1 (0.88)	0.84
History of status epilepticus	7 (2.60)	3 (2.65)	0.99
Provocative factors			
Feeling stressful	96 (35.69)	8 (7.08)	<0.01
Sleep deprivation	125 (46.47)	7 (6.19)	<0.01
Loud noise	6 (2.23)	0 (0)	0.18
Fatigue from works	23 (8.55)	0 (0)	<0.01
Bright light or blinking light	2 (1.12)	0 (0)	0.55
Weather change	6 (2.23)	0 (0)	0.18
Caffeine consumption	11 (4.09)	1 (0.88)	0.12
Alcohol consumption	26 (9.67)	2 (1.77)	<0.01
Menstruation	29 (10.78)	1 (0.88)	<0.01

**Table 2:** Univariate analysis of factors for seizure attack in persons with epilepsy.

Factors	Crude odds ratio	95% confidence interval	*p* value
Male	0.83	0.53–1.28	0.40
Age	0.98	0.96–0.99	<0.01
Single status	1.53	0.96–2.43	0.06
Complex partial seizure	2.23	1.38–3.58	<0.01
Feeling stressful	7.28	3.40–15.58	<0.01
Sleep deprivation	6.30	5.89–29.29	<0.01
Caffeine consumption	4.77	0.60–37.43	0.06
Alcohol consumption	5.93	1.38–25.45	0.02
Menstruation	13.53	1.82–100.60	0.01

**Table 3:** Multivariate analysis of factors for seizure attack in persons with epilepsy.

Factors	Adjusted odds ratio	95% confidence interval	*p* value
Male	0.89	0.52–1.52	0.68
Age	0.99	0.98–1.01	0.89
Single status	1.56	0.79–3.07	0.19
Complex partial seizure	1.87	1.09–3.23	0.02
Feeling stressful	2.97	1.29–6.86	0.01
Sleep deprivation	8.64	3.73–19.99	<0.01
Caffeine consumption	2.29	0.24–21.30	0.46
Alcohol consumption	6.76	1.44–31.78	0.01
Menstruation	6.26	0.76–51.08	0.08
